# Exploring preparedness transitions in medicine and pharmacy: a qualitative longitudinal study to inform multiprofessional learning opportunities

**DOI:** 10.1007/s10459-024-10372-w

**Published:** 2024-09-16

**Authors:** Ella Ottrey, Charlotte E. Rees, Caitlin Kemp, Kayley M. Lyons, Tina P. Brock, Michelle Leech, Lynn V. Monrouxe, Claire Palermo

**Affiliations:** 1 Monash Centre for Scholarship in Health Education (MCSHE), Faculty of Medicine, Nursing & Health Sciences, Monash University, Clayton, VIC Australia; 2 School of Health Sciences, College of Health, Medicine & Wellbeing, The University of Newcastle, Callaghan, NSW Australia; 3 Faculty of Pharmacy and Pharmaceutical Sciences, Monash University, Parkville, VIC Australia; 4 Centre for Digital Transformation of Health, Faculty of Medicine, Dentistry and Health Science, University of Melbourne, Melbourne, VIC Australia; 5 Centre for Collaborative Practice, University of Melbourne, Melbourne, VIC Australia; 6 Medicine Course, Faculty of Medicine, Nursing & Health Sciences, Monash University, Clayton, VIC Australia; 7 School of Health Sciences, Faculty of Medicine and Health, The University of Sydney, Camperdown, Sydney, NSW Australia

**Keywords:** Medicine, Pharmacy, Graduates, Preparedness, Transitions, Qualitative longitudinal research (QLR), Longitudinal qualitative research (LQR)

## Abstract

Despite extensive preparedness literature, existing studies fail to adequately explore healthcare graduates’ feelings of preparedness longitudinally across new graduate transition journeys, nor do they compare different healthcare professions to ascertain what opportunities exist for multiprofessional transition interventions. Therefore, this Australian study, underpinned by temporal theory, explores the preparedness transitions of medicine and pharmacy graduates. Our 6-month qualitative longitudinal study involved 12 medicine and 7 pharmacy learners after purposive sampling. They participated in an entrance interview before starting internship, longitudinal audio-diaries during their first three months of internship, and an exit interview. Framework analysis explored patterns in the data cross-sectionally and longitudinally for the whole cohort (thinking over time), with pen portraits illustrating individuals’ journeys (thinking through time). Preparedness and unpreparedness narratives involved practical skills and tasks, interpersonal skills, knowledge, and professional practice for medicine and pharmacy. However, narratives for practical skills and tasks, and professional practice were dominant amongst medicine graduates, while narratives for interpersonal skills and knowledge were dominant amongst pharmacy graduates. We found numerous cohort changes in feelings of preparedness over time, but the illustrative pen portraits demonstrated the complexities and nuances through time, including feelings of preparedness before internship becoming unpreparedness during internship (e.g., cannulas), improving preparedness through time (e.g., cover shifts), and persistent feelings of unpreparedness (e.g., patient interactions). While our cross-sectional findings are reasonably consistent with existing research, our comparative and longitudinal findings are novel. We recommend that educators build learners’ preparedness through uniprofessional transition interventions involving practical skills and tasks, and professional practice in medicine, and interpersonal skills and knowledge in pharmacy. More importantly, we recommend multiprofessional transition interventions for medicine and pharmacy learners before internship focusing on knowledge, and during internship focusing on practical skills and tasks.

**Supplementary Information:**

The online version contains supplementary material available at 10.1007/s10459-024-10372-w.

## Introduction

Preparedness for practice matters in healthcare. It matters to new graduates as they navigate exciting and daunting transitions into the world of healthcare work, with its associated responsibilities and identities. It matters to new graduates’ colleagues as they simultaneously rely on their contributions to the healthcare team, and serve to support, mentor, and manage these novice colleagues. And perhaps most importantly, preparedness matters to patients and their significant others, who expect to be treated with safety, dignity, and competence by new graduates. Therefore, considerable literature already exists outlining what healthcare graduates feel prepared or unprepared for. However, to date, this literature is largely uniprofessional and cross-sectional, meaning that crucial gaps remain in healthcare educators’ knowledge about the similarities and differences in preparedness between professions, and how preparedness changes through time. As such, educators may not fully realise the opportunities to develop multiprofessional transition to practice interventions, nor identify the ideal content and timing for these interventions. Therefore, this novel study extends the burgeoning preparedness literature by exploring comparatively graduates’ feelings of preparedness longitudinally across their final year student-new graduate transitions in medicine and pharmacy. This work enables us to provide recommendations for uniprofessional and multiprofessional transition interventions across new graduates’ journeys into and through internship.

### Preparedness across healthcare professions

Interestingly, preparedness has been conceptualised in multiple ways in the literature including experience, knowledge, confidence, resilience, short-term, and so on (Brennan et al., 2024; Ottrey et al., 2021). Considerable cross-sectional literature exists outlining the preparedness of uniprofessional groups for practice. A recent literature review revealed that 70–80% of medical graduates report feeling adequately prepared, but this was task-dependent (Padley et al., 2021). Regarding pharmacy interns, studies have reported diversity in preparedness for practice (see Table 1). A handful of studies have explored preparedness (sometimes focusing on preparedness for interprofessional practice) in final year students or new graduates from multidisciplinary groups, including: medicine, dentistry, nursing and/or midwifery, and/or allied health (Brennan et al., 2024; Ebert et al., 2014; Malau-Aduli et al., 2022; Merga, 2016; Walker et al., 2013). Together, these studies illustrate that final year students or new graduates are generally prepared for patient-centred practice, history-taking, examining patients, basic clinical skills, using guidelines, promoting patient safety and hygiene, and seeking support (Malau-Aduli et al., 2022; Walker et al., 2013). However, they illustrate under-preparedness for communicating with diverse people, clinical reasoning, prescribing, providing nutrition care, interprofessional teamworking, managing interpersonal conflict, recognising when to seek support, ward-specific knowledge, awareness of hospital policies and procedures, caseload and time management, clinical administration skills (e.g., audit, informatics, governance), high-risk patients and emergencies, applying theoretical knowledge to practice, teaching, coping with uncertainty, and stress management (Ebert et al., 2014; Malau-Aduli et al., 2022; Merga, 2016; Walker et al., 2013). On balance, preparedness for practice appeared similar across diverse professions, although some studies have started to illuminate differences (e.g., nursing graduates struggled to seek support and apply theory to practice, but medical graduates did not: Merga, 2016; Walker et al., 2013; Brennan et al., 2024). While these cross-sectional studies help to shed important light on healthcare graduates’ preparedness, numerous scholars have called for longitudinal research to further advance this important topic (e.g., Malau-Aduli et al., 2022; Monrouxe et al., 2017, 2018; Sumpradit et al., 2014). However, few longitudinal studies exist to date (Padley et al., 2021), and several only report cross-sectional findings (e.g., Lefroy et al., 2017; Monrouxe et al., 2018).

**Table 1:** Summary of preparedness for practice literature in medicine and pharmacy

	Medicine^*^	Pharmacy^**^
Prepared	History-taking Physical examinations Clinical skills (e.g., taking & recording observations, respiratory function tests, venepuncture, urine dipstick, blood cultures, electrocardiograms, intravenous cannulation, catheterisation, diagnosis & treatment planning for straightforward cases, writing discharge summaries) Ethics (e.g., patient consent)	Knowledge of conditions & treatment options Technical activities (e.g., dispensing & retail) Non-prescription & prescription activities (e.g., renewing existing prescriptions) Pharmacy regulation Patient care activities (e.g., dealing with anaphylactic reactions to vaccines, ambulatory care, counselling & medicines information roles)
Unprepared	Awareness of guidelines & protocols & accessing/applying existing knowledge Clinical skills (e.g., wound care & suturing, nasogastric tube placement, central venous catheter & chest drain insertion, clinical reasoning & diagnoses, treatment planning & prioritisation, prescribing, writing discharge letters, signing) Emergencies (e.g., responding to acutely unwell patients & knowing when to escalate) Communication with patients, their relatives, and colleagues (especially in mental health contexts) Interprofessional teamworking & disputes Protecting patients and improving care Error/safety incidents Complex ethical/legal issues (e.g., Do Not Attempt Resuscitation forms) Managing daily clinical workload in the time allotted, prioritisation, information overload & workplace stress Providing out-of-hours service Ward environment unfamiliarity	Practical application of knowledge in day-to-day patient management Dealing with practical drug management-related issues like adapting prescriptions & initiating smoking cessation therapy Decision-making, being in charge & accountability Communication with patients with mental health problems Humanistic skills (e.g., values and ethics) & relationship management Health promotion Multidisciplinary teamworking (incl. handling conflict) Health administration, pharmaceutical marketing & business management Adapting to the workplace & customer service

*Baten et al. (2018); Brinkman et al. (2018); Burridge et al. (2020); Cameron et al. (2014); Corfield et al. (2021); Hawkins et al. (2021); Lefroy et al. (2017); Michaelides et al. (2020); Monrouxe et al. (2017); Monrouxe et al. (2018); Monti et al. (2020); Padley et al. (2021)

**Chanakit et al. (2015); James and Cole (2016); Jee et al. (2017); Magola et al. (2018); Parmar et al. (2020); Rutter et al. (2013); Sumpradit et al. (2014); Waite et al. (2018)

### Temporal theory: preparedness across healthcare professions through and over time

Time is central to qualitative longitudinal research (QLR: Neale, 2018, 2019), and can be conceptualised in various ways (Rees & Ottrey, in press). In health-related QLR, time is often conceptualised as change, process, transition, and development (Audulv et al., 2022). Perhaps most relevant to the current study, time can be conceptualised as subjective, fluid and dynamic (i.e., thinking through time) or objective, linear and fixed (i.e., thinking over time: Audulv et al., 2022; Balmer & Richards, 2022; Balmer et al., 2021). Indeed, thinking *through* time enables researchers to focus on analysing experiences throughout a journey, including: “the stops and starts, detours, transitions and reversals of students’ progression” (Balmer et al., 2021, p. 1254). Conversely, thinking *over* time enables researchers to compare experiences at multiple timepoints, such as the start and end of rotations. Embedded within this fixed/fluid conceptualisation, time can also be considered as a snapshot in time or unfolding through time (called synchronic and diachronic respectively: Neale, 2018, 2019). Furthermore, researchers can orientate themselves to time across multiple planes including: (a) past, present and/or future (prospective-retrospective); and (b) short or long-term (intensive-extensive: Audulv et al., 2022; Balmer et al., 2021; Neale, 2018, 2019).

Four longitudinal studies have explored medical or pharmacy graduates’ preparedness temporally: either *through* or *over* time. Viewing time as fluid, Monrouxe et al. (2014) conducted a longitudinal audio-diary (LAD) study exploring medical graduates’ preparedness through time. However, three longitudinal questionnaire studies explored preparedness in medical or pharmacy graduates over time, viewing time as fixed (Chaou et al., 2021; Chow et al., 2022; Mak et al., 2013). These four studies demonstrate general improvements in competence and confidence across time, such as for history-taking, physical examinations, clinical reasoning, and communication (Chaou et al., 2021; Chow et al., 2022; Mak et al., 2013; Monrouxe et al., 2014). However, these studies also illustrate ongoing unpreparedness (e.g., for end-of-life care, mental state examinations, and respiratory function tests) despite transition interventions (Chow et al., 2022), and challenging circumstances generating feelings of unpreparedness for aspects that graduates had previously felt prepared for (e.g., being on-call, interprofessional teamworking, patient management, and self-directed learning: Chaou et al., 2021; Monrouxe et al., 2014). Finally, some cross-sectional and retrospective studies involving medical graduates have reported improvements in preparedness *over* time, such as responding to acutely unwell patients, carrying out clinical procedures, and coping with new clinical situations (Burridge et al., 2020; Walker et al., 2013).

### Critique of literature and study aim

The primary research outlined above typically involved descriptive, cross-sectional questionnaire surveys with uniprofessional graduates or educators. Some studies employed interviews with graduates or educators at one timepoint. The few multiprofessional studies conducted were also typically cross-sectional descriptive studies employing surveys or interviews/focus groups, and explored preparedness/readiness for clinical practice, including interprofessional practice. Additional criticisms include small sample (or sub-group) sizes and/or low response rates for questionnaires. Uniprofessional studies make it hard to understand the similarities and differences between new graduates’ preparedness, and thus what opportunities exist to develop multiprofessional transition interventions. Cross-sectional studies make it challenging to know how preparedness changes through time, and thus when multiprofessional transition interventions should be optimally timed. Therefore, this study—part of a broader research program on new graduate transitions amongst learners from dietetics, medicine, nursing, and pharmacy (Blair et al., 2023; Ottrey et al., 2021; Rees et al., 2022)—aims to explore medicine and pharmacy learners’ feelings of preparedness during their final year student-new graduate transition journeys (i.e., the months after graduation) to better understand what opportunities might exist for multiprofessional transition interventions. We sought to answer two research questions (RQs) based on temporal theory:

RQ1: What are the key areas of perceived preparedness and unpreparedness for medicine and pharmacy graduates, and how do they compare between the professions?

RQ2: How do feelings of preparedness change over and through the final year student-new graduate transition, and how do they compare between medicine and pharmacy graduates?

## Methods

### Study design and grand theories

**Fig. 1:**
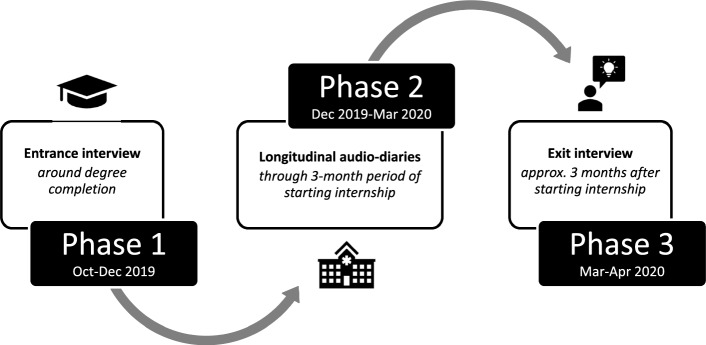
Visual representation of the data collection methods and their timing

This Australian study employs QLR (Neale, 2018; Neale & Flowerdew, 2003; SmithBattle et al., 2018; Vogl et al., 2018), grounded in social constructionism, valuing diversity in experiences and the construction of knowledge through social interaction (Burr, 2015; Rees et al., 2020). Indeed, our QLR was based on interpretivist philosophies privileging relativist ontology, subjectivist epistemology, and temporality of participants’ experiences. Our study design aligns with conceptualisations of time as fixed and fluid, synchronic and diachronic, prospective and retrospective, micro (i.e., individuals/small collectives), and intensive (i.e., short-term: Audulv et al., 2022; Balmer et al., 2021; Neale, 2018, 2019; Rees & Ottrey, in press). We elicited stories about preparedness from participants across three phases: (1) entrance interviews around degree completion before starting internship, (2) longitudinal audio-diaries (LADs) through a 3-month period entering the workforce as interns, and (3) exit interviews (Gordon et al., 2017, 2020; Monrouxe, 2009). See Fig. 1 for a visual representation of the data collection methods and their timing.

### Context

At the time of the study, this undergraduate medical degree, accredited by the Australian Medical Council, was a five-year program; with two pre-clinical years, followed by three years with approximately 560 days of hospital and community-based clinical placements (with Year 5 predominantly in hospitals). Most students began the program in Year 1, but approximately 25% took graduate entry into Year 2 (after completing biomedicine, science, physiotherapy, or pharmacy degrees). The learning modes were varied (i.e., lectures, seminars, workshops, tutorials, and simulation sessions), with interprofessional activities (with nursing and midwifery, nutrition science, and radiation science students) embedded in curriculum and assessment activities throughout the program. Transition interventions designed to support preparation for practice in Year 5 included ‘back to base’ days every 4–6 weeks and a ‘back to base’ week just prior to graduation. Medicine graduates then entered a paid internship year at an approved hospital with provisional registration. At the time of the study, internship involved five 10-week rotations through medicine, emergency medicine, surgery, and other specialties (e.g., mental health, neurology/stroke, palliative care), at metropolitan and/or rural/regional hospitals. After students submitted their rotation preferences, internships were coordinated through the Postgraduate Medical Council of Victoria. At the time of the study, this undergraduate pharmacy degree was comprised of four (predominantly pre-clinical) years accredited by the Australian Pharmacy Council. While most students began the program in Year 1, graduate entry into Year 3 was offered to those with science or biomedical science degrees. The teaching models were predominately lectures, tutorials and laboratory activities, with course content focusing primarily on foundational sciences. Students completed 60 days of professional experiential placement across Years 3 and 4. No specific multi- or interprofessional training or substantial transition interventions were included. Pharmacy graduates entered a one-year paid (clinical) internship program, operated by the university or another accredited internship provider (e.g., professional society), and undertaken at community pharmacies or in hospitals. At the time of the study, internship involved 1824 h of supervised practice, and could include rotations of various lengths through different settings (e.g., hospital dispensary, wards), in addition to assessments, workshops, and training sessions ahead of the Pharmacy Board examination. Both medicine and pharmacy interns secured full registration via the Australian Health Practitioner Regulation Agency on successful completion of their intern year.

### Sampling and recruitment

After securing ethics approval, we used purposive (i.e., maximum variation) sampling to recruit diverse final year students from four professions at an Australian university (Kitto et al., 2008). We wanted diversity in terms of students’ demographics (i.e., gender, ethnicity, and age), as well as professional affiliations (i.e., dietetics, medicine, nursing, and pharmacy). This paper focuses on medicine and pharmacy graduates only because: (a) both have provisional registration granted after graduation with full registration after successfully completing a 12-month internship; and (b) the increased task shifting and scope of practice for pharmacy (e.g., prescribing, vaccinating). We employed different recruitment methods, including email and in-person invitations, and snowballing. Our temporal sampling of final year students (before and during their early experiences of work as interns typically within hospitals but sometimes in a community setting) allowed us to track preparedness views and experiences through the university to workforce transition across a 6-month period. In total, 19 final year students participated in phases 1 and 2 (12 medicine, 7 pharmacy), with reasonable retention into phase 3 (15 participants: 9 medicine, 6 pharmacy). See Online Supplementary Table for a summary of participants’ involvement across the study. At the study outset, participants were predominately female (n = 16, 84%) and of Oceanian descent (n = 12, 63%; as defined by the Australian Bureau of Statistics, including Australian Peoples), with a median age of 23 years. Further participant demographics are reported elsewhere (Blair et al., 2023; Ottrey et al., 2021; Rees et al., 2022).

### Data collection

In the first phase, entrance interviews, we spoke to 19 participants in eight group interviews (5 medicine groups, 3 pharmacy groups; purposefully grouping participants by discipline) (Oct–Dec 2019). All interviews were conducted in-person and audio-recorded. Participants completed a personal details questionnaire. Then, led by a discussion guide to promote consistency between interviewers (CK, KML, EO), we asked participants to tell us what they thought about ‘preparedness for practice’ (Ottrey et al., 2021) and ‘transition’ (Rees et al., 2022). Employing narrative interviewing techniques (Riessman, 2008), we invited participants to share memorable stories of times when they felt prepared and unprepared for practice in their final year of study (see Appendix 1). When needed, we prompted for further information, such as when and where the scenario happened. Afterwards, we briefed participants on the second phase and invited their participation. Entrance interviews lasted on average 72 min (range 58–88), generating 9 h and 31 min of data.

All 19 participants (100%) took part in the second phase, where we used longitudinal audio-diaries (LADs) to gather preparedness stories weekly through a 3-month period during Dec 2019–Mar 2020 (Monrouxe, 2009). We provided a LAD guide, which prompted topics such as preparedness or unpreparedness experiences from that week, and their impacts (see Appendix 1). The LAD guide encouraged participants to share stories of preparedness and unpreparedness equally. Participants used their smartphones to audio-record their LADs and return them via email. We corresponded with participants weekly, sending LAD reminders, acknowledging each LAD returned, and asking follow-up questions. In total, we received 136 LADs, which lasted on average 8 min (range 2–22), amounting to 17 h and 10 min of data. LAD participation lasted on average 10 weeks (range 2–14), with participants submitting on average 7 LADs (range 2–12). See Online Supplementary Table for LAD participant details. LAD participants were invited to complete the third phase.

In the third phase, exit interviews, we spoke to 15 participants (79%) in six group interviews (4 medicine groups, 2 pharmacy groups) and one individual interview (medicine) (Mar–Apr 2020). We conducted the interviews in-person or via Zoom, depending on participants’ availability and preferences. We modelled our discussion guide on that used in the entrance interviews, with added emphasis on what had changed through time and why (i.e., their ‘long story’: see Appendix 1). Exit interviews lasted on average 69 min (range 53–86), generating 8 h and 3 min of data.

### Data analysis

We analysed our QLR data cross-sectionally (RQ1) and longitudinally (RQ2), as well as comparatively (both RQs) following the five-stage framework method (Ritchie & Spencer, 1994).

- *Stage 1, familiarisation*: Each team member independently reviewed two of 10 transcripts by listening to the relevant audio-recording while reading each transcript. We annotated the transcripts with our thoughts relating to the research questions, summarising our key points at the end of each transcript.
- *Stage 2, identifying the thematic framework*: All team members met to discuss key points, pulling these together into a preliminary coding framework. This was further developed, including contextual themes (e.g., narrative type, such as prepared story) and conceptual themes (e.g., narrative focus, such as communication), along with definitions and examples for each theme. Development of the coding framework was inductive and deductive, drawing on published research (Monrouxe et al., 2014, 2018), national competency standards (e.g., Pharamaceutical Society of Australia, 2016) and intern outcome statements (e.g., Australian Medical Council & Medical Board of Australia, 2013).
- *Stage 3, indexing*: Two team members (CK, EO) used the coding framework to code transcripts in NVivo (Version 12.2.0, QSR International). We first located narratives in participants’ talk, then coded the narrative type: prepared story, unprepared story, both (i.e., story with prepared and unprepared elements), or unclear. Next, we coded the narrative focus, using higher order and sub-themes. Sometimes narratives were coded to multiple foci. CK and EO met regularly to discuss coding progress and any challenges encountered, revising the coding framework as required.
- *Stage 4, charting*: We used matrix coding queries to explicate data patterns, such as dominant narrative types over and through time, and similarities and differences in narrative foci between medicine and pharmacy graduates. In this way, we identified and explored preparedness longitudinally (e.g., through time at the individual level, and over time at discipline and cohort levels) and cross-sectionally (e.g., by discipline). So, we viewed/treated time as both fluid and fixed (Balmer & Richards, 2022; Balmer et al., 2021). While we explored patterns through “credentialing counting” (i.e., frequencies and percentages helping us evidence our interpretations: Monrouxe & Rees, 2020, p. 186), we ‘closet’ (i.e., hide) frequencies and percentages when presenting our findings to avoid undermining our nuanced/complex qualitative meaning-making, which is possible if readers privileged quantities rather than qualities (Hannah & Lautsch, 2011; Monrouxe & Rees, 2020). Furthermore, we developed two pen portraits (reports for individual cases that: “successfully concentrate a large amount of longitudinal qualitative data into a focused account”: Sheard & Marsh, 2019, p. 2–3) to better understand and illustrate the longitudinal narratives from one medical intern and one pharmacy intern, chronologically documenting their transition journeys, and highlighting developments through time (Neale, 2018; Sheard & Marsh, 2019). Note that we selected these two participants because they were involved in all three study phases, and with rich data to illustrate their diverse experiences.
- *Stage 5, mapping and interpretation*: We interpreted our findings firstly by drawing upon published literature relating to preparedness, transitions, and QLR. We secondly interpreted our comparative findings to make sense of their implications for multiprofessional learning opportunities.

### Research team and reflexivity

All eight members of our authorship team for this paper have health/education-related backgrounds (3 dietetics, 2 pharmacy, 2 psychology, 1 medicine). Six have clinical experience, five are experienced educators, and three held teaching/coordinating roles in the degrees sampled during the study. The three team members responsible for participant recruitment and data collection (CK, KML, EO) had no prior relationships with participants. Undertaking a team reflexivity activity at the start of our research (to discuss our backgrounds, expertise, philosophical positioning, hopes and fears for the project) supported an understanding and appreciation of the clinical and educational diversity within our team, strengthening our ability to interpret and contextualise the findings (Barry et al., 1999).

## Results

We identified 424 narratives in participants’ data. Of these, 222 were coded as unprepared, and 160 as prepared. A further 37 contained both prepared and unprepared elements, and 5 were unclear. For clarity, in this paper we focus only on the 382 narratives that were coded as prepared or unprepared (except in the longitudinal pen portraits, where we synthesise and report data from two participants, irrespective of narrative type).

### RQ1: What are the key areas of perceived preparedness and unpreparedness for medicine and pharmacy graduates, and how do they compare between the professions?

**Fig. 2:**
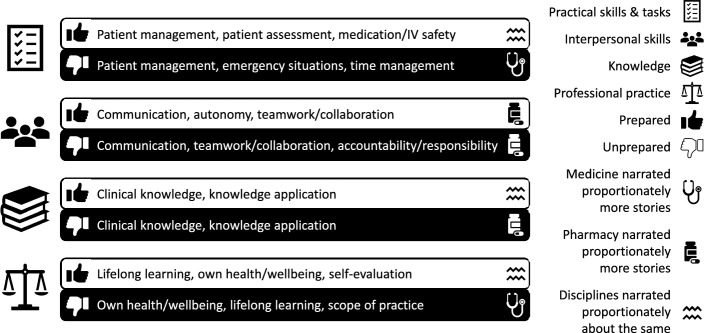
Visual representation of our RQ1 findings across the four higher order themes. This figure illustrates the four higher order themes (i.e., practical skills and tasks, interpersonal skills, knowledge, and professional practice) in medicine and pharmacy participants’ preparedness narratives. The thumbs up and thumbs down icons reflect the dominant sub-themes for which participants reported feeling prepared and unprepared for, respectively

Across the 382 narratives, participants’ stories sometimes focused on multiple higher order themes, which were: practical skills and tasks, interpersonal skills, knowledge, and professional practice. Despite guidance to share prepared and unprepared stories equally, of the 229 stories recounted by medicine participants, there were substantially more unprepared stories than prepared stories. However, of the 153 stories narrated by pharmacy participants, there were similar numbers of unprepared and prepared stories. A synthesis of our RQ1 findings across the four higher order themes is visually represented in Fig. 2.

#### Feelings of preparedness for practical skills and tasks

Participants narrated more unprepared stories about practical skills and tasks than prepared stories. Unprepared stories were primarily about patient management, such as difficulties with medications, discharge summaries, suboptimal handover, patients with multiple comorbidities, and management plans. Emergency situations, including Medical Emergency Team calls, and deteriorating or aggressive patients were also challenging for new graduates. Unprepared time management stories included struggling with busy/fast-paced environments, triaging/prioritising tasks, working overtime, and efficiency. This is illustrated by a female pharmacy graduate, who describes her unpreparedness for efficiently completing medication reconciliations in one of her LADs:

“… I had to do the med recs [medication reconciliations]… when you haven’t been on a ward before… when you go through the patient notes, a lot of it is medical abbreviation and jargon… and so it takes me a little while to research it, and then put it into context. And then going through notes after notes. It’s a very time-consuming process…” (FG17P3P1F).

Prepared stories commonly focused on patient management, such as comfort with reviewing patients, medications/fluids, discharge summaries, management plans, writing referrals, and completing tasks/procedures. For example, a female medicine graduate describes her preparedness for catheterising a patient in one of her LADs:

“… this co-intern… they left… before they got a chance to do it [catheterise a patient with urinary retention]. So, I ended up putting the catheter in… I could do this procedure without needing any supervision, without needing to ask for anything. And it went well.” (FG18M5P2F).

Prepared stories about patient assessment included ease with history-taking, performing physical examinations, developing diagnoses and management plans, and reporting back to seniors. Prepared stories were also about medication/IV safety, such as evaluating medication appropriateness, identifying drug interactions and errors, as well as advising on optimal medication timing/duration. Interestingly, medical graduates narrated proportionately more unprepared stories about practical skills and tasks than pharmacy graduates.

#### Feelings of preparedness for interpersonal skills

Participants recounted more prepared stories about interpersonal skills than unprepared stories. Prepared stories were typically about communication, including comfort with intra/interprofessional interactions (e.g., contacting prescribers, making referrals), completing written documentation (e.g., progress notes, medication charts, discharge summaries), and interviewing/counselling patients and family members. Prepared autonomy stories also included doing activities independently (e.g., making decisions, solving problems). Prepared stories about teamwork/collaboration included ease with building relationships, supporting new team members, requesting/accepting support, and assisting colleagues. For example, one female medicine graduate explains in her entrance interview how she felt prepared to contribute to the workload of the medical team in one of her final year rotations:

“I had a rotation in gen med… There’d be so much paperwork… me and [fellow medical student] had to step up and kind of, help out with the workload of the team, else we would never finish... having the chance to actually do stuff, was actually probably the most important thing [for helping feelings of preparedness]…” (FG11M2P2F).

Unprepared stories predominately focused on communication, including challenges with interviewing/counselling patients and family members (e.g., non-English speakers, breaking bad news, emotional individuals, conflict management), completing written documentation, interacting with seniors (e.g., case presentations, responding to criticism), and intra/interprofessional interactions. Unprepared stories about teamwork/collaboration included difficulties getting along with others, negotiating hierarchies, managing differing opinions, task delegation, and advice/information seeking. For example, one female pharmacy graduate describes in her exit interview her unpreparedness for challenging a medical prescriber’s prescription:

“I took the script down to the pharmacy, and I started dispensing it, and I was kind of like, ‘hmm, this doesn’t seem right’… I said to the pharmacist in the dispensary… ‘Oh, they’ve done this, but they actually need to do this.’ And they’re like, ‘Well, in that case, you need to contact the prescriber.’ I was like, ‘Oh, no, I don’t want to do that.’ [laughs]. And they were like, ‘Well, you’ve got to’…” (FG15P1P1F).

Unprepared accountability/responsibility stories included struggling with running clinics, writing/dictating letters, answering patients’/colleagues’ questions, and escalating care. Interestingly, pharmacy graduates narrated proportionately more prepared *and* unprepared stories about interpersonal skills than medical graduates.

#### Feelings of preparedness for knowledge

There were slightly more unprepared stories narrated about knowledge than prepared stories. Unprepared stories primarily focused on clinical knowledge, including challenges with pharmacy internship exam preparation, feeling pressured to have the right answer, encountering unfamiliar scenarios, and lacking knowledge about tests, treatments, and devices. Unprepared knowledge application stories included mind blanks when treating/counselling patients and realising the need to practise skills/tasks and build experience. A female pharmacy graduate describes her unpreparedness in one of her LADs regarding her knowledge of a specific antidepressant:

“[My preceptor] quizzed me on an antidepressant… It was a simple antidepressant… and I thought I knew it. Clearly, I didn’t know as well as I should have. So, I’ve still got a long way to go.” (FG16P2P1F).

Prepared stories were commonly about clinical knowledge, such as comfort with learning/retaining content about conditions, procedures, and medications, answering questions, knowing when to ask for help, and locating information. A female medicine graduate explains in one of her LADs her preparedness for finding information:

“The other thing that I think I’ve been prepared for well is when I don’t know what to do, I can look it up very easily. And we’ve been given a whole bunch of resources that we’ve used throughout med school… that’s been really good to know exactly where to go and how to look something up.” (FG10M1P2F).

Prepared stories about knowledge application included ease with drawing on different information to develop/initiate management plans, identifying medication errors and suggesting alternatives, and recalling information to provide advice. Intriguingly, pharmacy graduates narrated proportionately more unprepared stories about knowledge than medical graduates.

#### Feelings of preparedness for professional practice

There were slightly more unprepared stories recounted about professional practice than prepared stories. Unprepared stories were predominately about participants’ own health/wellbeing, including difficulties adjusting to full-time work as interns (e.g., night shifts and weekends), establishing routines, maintaining work/life/study balance, taking breaks and personal leave, and managing emotions, exhaustion, and stress. Unprepared lifelong learning stories included challenges with identifying learning goals, keeping abreast of industry news, and exam preparation. Unprepared stories were also about scope of practice, including understanding local prescribing protocols and when to refer to other disciplines. This is illustrated by a male medicine graduate in his exit interview:

“I guess scope of practice is something that I’m still trying to determine… when it’s a good idea to page or request an assessment from other professionals… when a consultant especially asks a task to be done by you, I find it a bit difficult to say, ‘Is it alright if I hand it over to someone else?’...” (FG11M2P1M).

Prepared stories typically focused on lifelong learning, such as comfort with internship training, exam preparation, accepting/learning from mistakes, accessing resources, and drawing on others’ expertise to develop knowledge/skills. Prepared stories about participants’ own health/wellbeing included ease with establishing routines, undertaking self-care activities, and maintaining work/life balance. Prepared self-evaluation stories included mid/end of rotation assessments, participants evaluating their own emotional states and impacts, and critically reflecting on their practice. This is illustrated by a female pharmacy graduate in one of her LADs:

“And other aspects that I enjoyed this week was when I was with more organised pharmacists in the dispensary, and they gave me the opportunity to counsel patients… when I finish counselling, I can reflect back on myself, my performance, and see areas that I could improve on… then when I speak to the next patient, I just try and incorporate the things that I thought I was missing...” (FG16P2P2F).

Interestingly, medical graduates narrated proportionately more unprepared stories about professional practice than pharmacy graduates.

### RQ2: How do feelings of preparedness change over and through the final year student-new graduate transition, and how do they compare between medicine and pharmacy graduates?

**Fig. 3:**
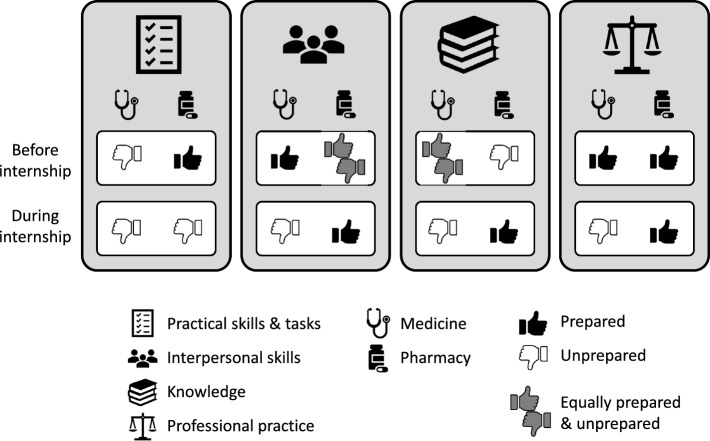
Visual representation of our RQ2 cohort findings over time. This figure illustrates the dominant narrative types (i.e., prepared = thumbs up or unprepared = thumbs down) for each of the four higher order themes, by profession and time-period. Note that ‘Before internship’ reflects data collected in Phase 1, while ‘During internship’ encompasses data collected in Phases 2 and 3

Across the 382 narratives, participants reported substantially fewer narratives before starting internship, compared to during internship. Before internship, narratives were about interpersonal skills, knowledge, practical skills and tasks, and professional practice. Narratives reported during internship commonly focused on practical skills and tasks, interpersonal skills, knowledge, and professional practice. While medicine participants recounted roughly equal numbers of unprepared and prepared stories before internship, there were substantially more unprepared stories during internship. They narrated more prepared stories about interpersonal skills and professional practice before internship, but more unprepared stories for these two themes during internship. They narrated equal numbers of prepared and unprepared stories about knowledge before internship, but this shifted to more unprepared stories during internship. They narrated more unprepared stories about practical skills and tasks before and during internship. Interestingly, pharmacy participants recounted roughly equal numbers of unprepared and prepared stories before and during internship. They narrated more prepared stories about practical skills and tasks, and professional practice before internship. While this positive trend continued during internship for professional practice, the dominant narrative type for practical skills and tasks changed to unprepared. They narrated equal numbers of prepared and unprepared stories about interpersonal skills before internship, but this shifted to more prepared stories during internship. Also showing a positive trend was preparedness for knowledge, where the dominant narrative type changed from unprepared before internship, to prepared during internship. A synthesis of our RQ2 cohort findings over time is visually represented in Fig. 3.

#### Changes over time: cohort patterns

In terms of practical skills and tasks, participants consistently recounted more unprepared stories over time. Although unprepared stories before internship commonly focused on patient assessment, patient management, and time management, during internship, they were often about patient management, time management, and emergency situations. Prepared stories before internship were about patient management and patient assessment, yet during internship, they centred on patient management, medication/IV safety, patient assessment, and workplace systems like comfort with electronic medical records, incident reporting, and human resources. Only medical students narrated unprepared stories about practical skills and tasks before internship. Furthermore, medical graduates narrated proportionately more unprepared stories about practical skills and tasks during internship than pharmacy graduates. Pharmacy graduates’ unprepared stories about practical skills and tasks increased over time. Regarding interpersonal skills, participants consistently narrated more prepared stories over time. Prepared stories before internship predominately focused on communication, teamwork/collaboration, and accountability/responsibility. During internship, preparedness for communication and teamwork/collaboration remained common, with the addition of autonomy. While unprepared stories before internship were mostly about communication, accountability/ responsibility, and therapeutic relationships, during internship, they focused on communication, teamwork/collaboration, and accountability/responsibility. Before internship, medical students narrated proportionately fewer unprepared stories about interpersonal skills than pharmacy students. While this pattern continued during internship, the difference was less marked. Concerning knowledge, participants consistently reported more unprepared stories over time. Unprepared stories before and during internship were primarily about clinical knowledge and knowledge application. Similarly, prepared stories before and during internship were typically about clinical knowledge and knowledge application. Before internship, medical students narrated proportionately fewer unprepared stories about knowledge than pharmacy students. While this pattern continued during internship, the difference was less marked. Finally, relating to professional practice, participants narrated more prepared stories before internship, but more unprepared stories during internship. While prepared stories before internship were about participants’ own health/wellbeing, lifelong learning, and scope of practice, during internship, they centred on self-evaluation, lifelong learning, and participants’ own health/wellbeing. Although unprepared stories before internship were about participants’ own health/wellbeing, and lifelong learning, during internship, they focused on participants’ own health/wellbeing, scope of practice, safe practice, and lifelong learning. Only medical students narrated unprepared stories about professional practice before internship. Furthermore, medical graduates narrated proportionately more unprepared stories about professional practice during internship than pharmacy graduates. Pharmacy graduates’ unprepared stories about professional practice increased over time.

#### Changes through time: illustrative pen portraits

Pen portraits are now presented to illustrate two longitudinal cases (one medicine, one pharmacy). The first case is that of Josie (pseudonym), a female medical graduate (Box 1). She participated in an entrance interview, 10 LADs through an 11-week period, and an exit interview, providing 3 h and 58 min of data (weeks 0–23). As a final year student, Josie talked about her placements in rural and metropolitan health services, including in an emergency department. Upon graduation, she began her internship with a 10-week general surgical rotation in a metropolitan health service, before moving to a general medical rotation at a different hospital within the same health service. Josie’s exit interview took place approximately three weeks into her second rotation. The second case is that of Hannah (pseudonym), a female pharmacy graduate (Box 2). She participated in an entrance interview, 12 LADs through an 11-week period, and an exit interview, culminating in 4 h and 44 min of data (weeks 0–19). As a final year student, Hannah talked about her placements in community and hospital settings, including on general medicine and renal wards. Upon graduation, she started her internship with a 5-week rotation on a general medical ward in a regional health service. She then moved to the hospital dispensary for a short time, before rotating to a surgical ward with the same health service. Hannah’s exit interview took place approximately four weeks into her time on the surgical ward.

**Box 1:** Pen portrait for Josie (FG11M2P3F), a female graduate from medicine

Before internship (Data from entrance interview and LAD1: weeks 0 to 10): In her narratives recounted before starting work as an intern, Josie spoke of feeling prepared for some practical aspects of her role, such as conducting patient interviews [interpersonal skills], as well as examining patients, and procedures like inserting cannulas [practical skills and tasks]. However, she identified feeling unprepared for practical skills and tasks such as simulated emergencies, and knowledge, which had been a longstanding theme throughout her degree: “I’m happy to just look things up, or just fudge my way through, because I’ve done it for five years” (week 0)	
During internship (Data from LADs2-10 and exit interview: weeks 11 to 23): Commencing her internship on a surgery rotation in a metropolitan health service, Josie described mixed feelings about preparedness for practical skills and tasks such as inserting cannulas and booking procedures. However, she also reported feeling unprepared for knowing processes and systems such as ordering tests: “I don’t think I really realised how unprepared I was to just start working by myself in a system that I don’t really understand” (week 12). And she reported feeling unprepared for managing in a busy environment, triaging/prioritising tasks, working overtime, and completing tasks efficiently. She consistently narrated feelings of unpreparedness regarding practical skills like inserting catheters, as well as unexpected events like doing stitches in theatre: “that was a moment that I was wholly unprepared for… [I was] so shocked that I was even put into that position” (week 18). Regarding interpersonal skills, she narrates being prepared for getting along with some surgical seniors, but unprepared for responding to criticism and handling team changes, for example, being relied on when new registrars started, and needing to cover for her co-intern on leave. However, through time, Josie reported shifting from feeling unprepared for some aspects of professional practice such as managing cover shifts (e.g., “nobody really warned me about what cover shift was going to be like”: week 12) to feeling prepared, where, for example, she covered all patients across multiple surgical teams and wards from 1–9 pm. For example, in her narratives about her first few cover shifts, Josie described feeling overwhelmed and confused, which was compounded by her unfamiliarity with patients, and a lack of understanding about her role for cover shifts: “I went in not really knowing what exactly… I was expected to do” (week 12). In contrast, she described her final cover shift in surgery as: “amazing” (week 21); a feeling that was bolstered by now knowing how the system worked, and being able to complete her tasks with ease: “I just smashed jobs all night” (week 21)

**Box 2:** Pen portrait for Hannah (FG16P2P2F), a female graduate from pharmacy

Before internship (Data from entrance interview and LAD1: weeks 0 to 6): In her narratives before starting work as an intern, Hannah talked about knowledge as an area she felt prepared for, such as identifying errors in medication charts. However, she more readily recounted feelings of unpreparedness, especially regarding her relationships with patients and doctors [interpersonal skills]. She described feeling pressure when counselling patients about their medications (e.g., “in that moment, I was really stressed”: week 0), and identified needing to strengthen her advocacy skills when communicating with “difficult patients” (week 6). This was also reflected in her interactions with doctors, where she admitted being reluctant to contact them, such as to request prescriptions. She talked about feeling like she needed to have the right answer, and worried about saying the wrong thing (“you’ve got to go to the doctor with the right answer the first time around”: week 0)	
During internship (Data from LADs2-12 and exit interview: weeks 7 to 19): Commencing her internship on a hospital ward in a regional health service, Hannah communicated her mixed feelings of preparedness for practical skills and tasks like writing on medication history forms, conducting medication reconciliations, and identifying medication errors, while feeling unprepared to use some devices such as an insulin pen. Regarding interpersonal skills, she describes feeling prepared speaking with pharmacies to get dispensing histories, liaising with her preceptor about patient admissions, taking medication histories, and advising patients: “I feel like I’m starting to make a few clinical interventions that I feel proud to have made" (week 10). However, she narrates unpreparedness for taking an accurate medication history from a challenging patient (and where the dispensing history was unavailable from the pharmacy), and for dealing with conflict with doctors. For example, she is advised: “you shouldn’t just agree with the doctor just because they’re the doctor” (week 17). She also reports unpreparedness for interacting with emotional patients and their caregivers, although she explained that her: "empathy towards the patients and their families are improving” (week 9). She also narrates unpreparedness for working with a new supervising pharmacist, and her challenges interacting with medical interns, particularly in terms of answering their questions [knowledge]: “I get really nervous when they speak to me because I really don’t want to give them the wrong answer” (week 10). She also narrates being unprepared for other aspects of knowledge, such as feeling: “really silly when I don’t know an answer” (week 11). Through time, Hannah narrated a shift from being unprepared for independence [professional practice] to growing independence: “I’m learning to deal with the independence a little bit better” (week 17). She also reported a shift from unpreparedness for identifying and communicating medication errors/problems with medical prescribers, to preparedness across her internship on the hospital ward [interpersonal skills and knowledge]. Early on, she describes this as: “really intimidating” (week 9), largely because she was: “worried about what they were going to say” (week 9). However, as her experience grows, so too does her comfort in speaking to doctors about medication problems to get them fixed for the benefit of patients: “communicating with healthcare [laughs] professionals is what I’ve improved in” (week 19)	

## Discussion

This comparative study explores medical and pharmacy learners’ perceptions of preparedness and unpreparedness, and how these feelings change over the final year student-new graduate transition. Our comparative analysis also provides insights into opportunities for multiprofessional learning before and during new graduate transitions. In this discussion, we first summarise our key findings and compare these with existing literature and theory, especially illustrating where our study extends existing research. We then outline the methodological strengths and limitations of the study, before concluding with the implications of the findings for further research and educational practice.

### Summary of key findings and comparison with existing literature and temporal theory

Regarding RQ1, several findings were consistent with existing literature: (a) participants’ examples of preparedness and unpreparedness involving practical skills and tasks, interpersonal skills, and knowledge (e.g., Monrouxe et al., 2017; Padley et al., 2021); (b) unprepared stories dominating participants’ narratives involving practical skills and tasks such as patient management, time management, and emergency situations (Brinkman et al., 2018; Burridge et al., 2020; James & Cole, 2016); (c) prepared stories dominating participants’ narratives about interpersonal skills such as communication, teamwork/collaboration, and autonomy (Lefroy et al., 2017; Monrouxe et al., 2018); (d) us coding slightly more unprepared stories involving knowledge like clinical knowledge and knowledge application (Malau-Aduli et al., 2022; Merga, 2016; Rutter et al., 2013); and (e) us coding slightly more unprepared stories involving professional practice such as own health/wellbeing, scope of practice, and lifelong learning (Baten et al., 2018; Lundin et al., 2018; Merga, 2016; Monti et al., 2020).

However, our findings for RQ1 extend this literature in several important ways. Firstly, we found that unprepared stories involving practical skills and tasks were especially dominant in medical graduates’ data. Their preoccupation with practical skills and tasks might reflect a broader scope of practice requiring them to enact (and worry about) a wider range of practical skills and tasks than pharmacy graduates. Secondly, we found that prepared and unprepared stories involving interpersonal skills were particularly dominant in pharmacy graduates’ data, suggesting that they were especially preoccupied with their preparedness for interpersonal skills. This preoccupation may reflect this cohort’s traditional knowledge-focused pharmacy curriculum, with only 60 days of experiential placements, before the pharmacy curriculum’s transformation to become more experiential and skills-based including communication and teamworking (Forrester et al., 2023). Thirdly, we found that unprepared stories involving knowledge were especially dominant in pharmacy graduates’ data. Their preoccupation with knowledge may reflect the knowledge-focused instruction and assessment that was dominant in their program at that time. It may also reflect pharmacy graduates’ anxieties about causing harm through medication errors, or inaccurate medicines information provided to patients and doctors. Finally, we found that unprepared stories involving professional practice were especially dominant in medical graduates’ data. Their preoccupation with professional practice might reflect their regular night/weekend working affecting their home life more than pharmacy graduates, as well as their challenges with patient management in terms of breaking bad news and death and dying, affecting their coping.

Regarding RQ2, comparing learner experiences before and during internship and thus conceptualising time as fixed and synchronic (Audulv et al., 2022; Balmer & Richards, 2022; Balmer et al., 2021), we found some cohort/discipline evidence of apparent stability *over* time, as has been found in existing literature (e.g., Chaou et al., 2021; Chow et al., 2022; Mak et al., 2013). In our study sample, we found that unprepared stories involving practical skills and tasks dominated before and during internship for medical participants, and prepared stories about professional practice dominated before and during internship for pharmacy participants. However, we also found cohort/discipline evidence of changes over time, consistent with existing literature (e.g., Chaou et al., 2021; Chow et al., 2022; Mak et al., 2013). In our study sample, while prepared stories were dominant in medical students’ data before internship, unprepared stories dominated during internship, illustrating that medical graduates were especially preoccupied with their unpreparedness for interpersonal skills, knowledge, and professional practice during internship. Conversely, unprepared stories (e.g., involving knowledge, interpersonal skills) were dominant in pharmacy students’ data before internship, yet prepared stories dominated during internship. Furthermore, while prepared stories dominated in pharmacy students’ narratives about practical skills and tasks before internship, unprepared stories dominated during internship.

However, conceptualising time as fluid and diachronic, we found in our two pen portraits changes *through* time; with evidence of the complexities and nuances of preparedness shifting through time, consistent with one previous UK study of junior doctors published in grey literature (Monrouxe et al., 2014). Bringing novelty to the literature, we identified examples of Josie (the medical graduate) and Hannah (the pharmacy graduate) feeling prepared before internship, but then unprepared during internship (e.g., inserting cannulas for Josie, knowledge for Hannah), examples of improved feelings of preparedness during the first three months of work as interns (e.g., cover shifts for Josie, interpersonal skills with colleagues for Hannah), and examples of persistent feelings of unpreparedness over that same time period (e.g., knowledge for Josie, interpersonal skills with patients for Hannah). Indeed, through the entrance/exit interviews and longitudinal audio-diaries, as well as the presentation of in-depth longitudinal pen portraits, we were able to witness the unfolding of Josie and Hannah’s preparedness journeys as they orientated themselves to past, present and future (prospective-retrospective) over a relatively short but intensive time-period (Audulv et al., 2022; Balmer et al., 2021; Neale, 2018, 2019). Interestingly, this conceptualisation of time as diachronic, subjective, fluid and dynamic (Audulv et al., 2022; Balmer & Richards, 2022; Balmer et al., 2021; Neale, 2018, 2019), clearly illustrated preparedness journeys as non-linear with “detours” and “reversals” mentioned by Balmer et al. (2021, p. 1254).

### Methodological strengths and challenges

We collected over 34 h of audio data with 19 medicine and pharmacy participants. Our sample had adequate information power given our narrow study aim (exploring preparedness during the final year student-new graduate transition), dense sample specificity (purposive sampling of final year students from two professions), application of established theory across our broader research program (Multiple and Multidimensional Transitions theory: Jindal-Snape, 2016; Rees et al., in press), high-quality interview dialogue (strong researcher-participant rapport supporting the collection of relevant and rich data), and our in-depth analysis strategy (cross-sectional and longitudinal comparative narrative analyses: Malterud et al., 2016). We experienced minimal attrition (four participants did not complete an exit interview), by using various engagement strategies including sending regular LAD reminders, acknowledging LAD receipt, providing reassuring feedback on LADs, returning LAD transcripts to participants, and providing $10 gift cards for each study phase completed. We also conducted a rigorous and reflexive team-based analysis of voluminous qualitative data, employing NVivo, which helped us to make sense of our data (the whole dataset, as well as individual cases) cross-sectionally and longitudinally.

However, our study is not without its challenges, and these must be considered before drawing conclusions. First, although our findings are largely supportive of existing international research, our study was conducted in one Australian university only, so our findings (and study implications) may lack transferability to non-Australian contexts with different models of healthcare education and service delivery. Second, given our funding constraints, we collected data over a relatively modest time-period (6-months); shorter than other longitudinal studies (e.g., Chaou et al., 2021; Mak et al., 2013; Monrouxe et al., 2014), and the time-periods suggested for graduates to feel prepared (e.g., Burridge et al., 2020; Walker et al., 2013). However, timeframe and tempo are often inter-connected in QLR (Rees & Ottrey, in press), so the relatively short duration is partly compensated for by the intensive weekly tempo of data collection for LADs. Plus, this study fills an important gap in the literature on early transitions into clinical practice in pharmacy (Brennan et al., 2024). Furthermore, our participants mostly identified as female and Oceanian, so our findings may not represent the experiences of those identifying as male or from an ethnic minority. Finally, our QLR findings outline patterns of dominance in participants’ narratives, which are not to be confused with prevalence of preparedness or unpreparedness across time. Indeed, despite us asking participants for equal numbers of prepared and unprepared stories across their transitions, we know that emotional experiences are more likely to be remembered and narrated (Rees et al. 2013), potentially accounting for the dominance in unprepared narratives across our study. However, this is an important finding in of itself, as we can see clearly in our QLR findings what issues students are most preoccupied with in terms of their preparedness and when, suggesting when they may be most receptive to transition interventions (and on what topics).

### Study implications

Given our study challenges, we recommend that further research is conducted at non-Australian universities (representing different models of healthcare education and service delivery) to establish the transferability of our findings to wider-ranging contexts. We also encourage researchers to conduct QLR across longer study durations to track the ebbs and flows of preparedness for practice across the internship year, and into full registration. Finally, further research should encourage the participation of students and graduates representing demographics underrepresented in this study (e.g., those identifying as male, and culturally and linguistically diverse professionals).

Regarding educational implications, this study set out to understand what (if any) opportunities exist to develop multiprofessional transition to practice interventions, when these interventions should be optimally timed, and on what topics. In Table 2, we outline recommendations for educators based on our findings. While the cross-sectional findings for RQ1 imply that uniprofessional transition interventions could be preferred (because medical and pharmacy learners are preoccupied with unpreparedness for different things), our longitudinal findings for RQ2 clearly indicate opportunities for multiprofessional transition interventions in final year and during the first few months of work as interns.

**Table 2:** Recommendations for educators

Key findings for RQs	Educational implications
*RQ1:* Medical graduates were particularly preoccupied with their unpreparedness for practical skills and tasks, and professional practice Pharmacy graduates were especially concerned with their unpreparedness for interpersonal skills and knowledge	*Uniprofessional transition interventions:* Medical educators should focus on building medical learners’ confidence and competence in transition interventions for practical skills and tasks, and professional practice Pharmacy educators should focus on growing pharmacy learners’ confidence and competence in transition interventions for interpersonal skills and knowledge
*RQ2:* Medical students were particularly preoccupied by their unpreparedness for practical skills and tasks, and knowledge before and during internship, and unpreparedness for interpersonal skills and professional practice during internship Pharmacy students were particularly preoccupied with their unpreparedness for interpersonal skills and knowledge before internship, and their practical skills and tasks during internship	*Multiprofessional transition interventions:* Educators should develop, implement, and evaluate multiprofessional transition interventions for medical and pharmacy interns: before internship focusing on knowledge, including clinical knowledge and knowledge application (e.g., medicines knowledge, clinical reasoning); during the first three months of work as interns focusing on practical skills and tasks, including patient management (e.g., medications, discharge summaries, handovers, patients with multiple comorbidities), time management (e.g., working efficiently in busy/fast-paced environments, triaging/prioritising tasks), and emergency situations (e.g., deteriorating patients)

## Electronic supplementary material

Below is the link to the electronic supplementary material.

Supplementary file1 (DOCX 16 kb)

## Data Availability

We do not have ethics approval to share our data openly, in order to protect the privacy of our study participants.

## Appendix 1 Illustrations of interview questions and audio-diary prompts^*^

**Table:**

Entrance interview	LAD prompts	Exit interviews
Can you share with me any memorable experiences from this year where you felt prepared for practice. Can you tell me a story in as much detail as possible? Prompts: What happened, in as much detail as you can remember? Where, when, who was involved, etc. What did you do? What was the reasoning behind what you did? What were you thinking at the time? What did you feel at the time? What helped your preparedness in that scenario? Is there anything that could have helped you to feel even more prepared for practice, and why? Probe: What are the emotional, psychological and social impacts of this memorable experience from your final year, and why? Can you share with me any memorable experiences from this year where you felt unprepared for practice? Prompts: *As above, modifying the last two prompts as follows:* What hindered your preparedness in that scenario? Is there anything that could have helped you to feel more prepared for practice, and why? Probe: *As above*	Please share with us an experience from your workplace this week that relates to preparedness/unpreparedness for practice. If you have had multiple experiences this week, please share with us your most memorable positive experience and/or your most memorable negative experience For each audio diary, you might like to use these questions as a guide: Tell us of a time this week when you felt prepared for practice and also a time when you felt less prepared: When and where did the events occur and who else was present? What happened? What did you do? What was the reasoning behind what you did? What were you thinking at the time? What did you feel at the time? How might your preparedness/unpreparedness for practice have influenced this experience and why? If not, why not? Any other comments you want to make about your workplace experiences and preparedness/unpreparedness for practice?	Have you had any other memorable experiences, since your last audio diary, that you haven’t yet shared? Can you share with me any memorable experiences from the past 4 months where you felt prepared for practice? Prompts: What happened, in as much detail as you can remember? Where, when, who was involved, etc. What did you do? What was the reasoning behind what you did? What were you thinking at the time? What did you feel at the time? What helped your preparedness in that scenario? Is there anything from your final year that could have helped you to feel even more prepared for practice, and why? Can you share with me any memorable experiences from the past 4 months where you felt unprepared for practice? Prompts: *As above, modifying the last two prompts as follows:* What hindered your preparedness in that scenario? Is there anything from your final year that could have helped you to feel more prepared for practice, and why? Now I would like to turn your attention to your long story. I hope you have had a chance to skim your audio diaries. Let’s think about preparedness for practice: How have your thoughts about this changed (or not) over time, and why? Prompts: For positive or negative? I noticed initially you felt unprepared for X, later it seemed like you nailed that. How did that feel for you?

*We only include questions and prompts here relevant to learners’ preparedness for practice experiences focused on in this paper; questions/prompts relevant to conceptualisations of transitions and preparedness, and broader transition experiences are published elsewhere (Ottrey et al., 2021; Rees et al., 2022; in press). Please contact the corresponding author to request copies of the full discussion and LAD guides.
