# Supplementary material for: Exploring preparedness transitions in medicine and pharmacy: a qualitative longitudinal study to inform multiprofessional learning opportunities
Source: Adv Health Sci Educ Theory Pract. 2024 Sep 16;30(3):711–34. doi: 10.1007/s10459-024-10372-w (PMC12119723; doi:10.1007/s10459-024-10372-w)
Supplement: Supplementary file 1 — Supplementary file1 (DOCX 16 kb) [file 10459_2024_10372_MOESM1_ESM.docx]

**Online Supplementary Table. Summary of participants’ involvement across the QLR study**

| **Participant** | **Profession** | **Participated in Phase 1 (entrance interview)** | **Participated in Phase 2 (number of LADs provided)** | **Participated in Phase 3 (exit interview)** |
| --- | --- | --- | --- | --- |
| FG10M1P1M | Medicine | Yes | 4 | No |
| FG10M1P2F | Medicine | Yes | 8 | Yes |
| FG11M2P1M | Medicine | Yes | 3 | Yes |
| FG11M2P2F | Medicine | Yes | 10 | Yes |
| FG11M2P3F* | Medicine | Yes | 10 | Yes |
| FG12M3P1F | Medicine | Yes | 2 | No |
| FG12M3P2M | Medicine | Yes | 11 | Yes |
| FG13M4P1F | Medicine | Yes | 4 | No |
| FG13M4P2F | Medicine | Yes | 10 | Yes |
| FG15P1P1F | Pharmacy | Yes | 7 | Yes |
| FG15P1P2F | Pharmacy | Yes | 9 | Yes |
| FG15P1P3F | Pharmacy | Yes | 7 | Yes |
| FG16P2P1F | Pharmacy | Yes | 8 | Yes |
| FG16P2P2F* | Pharmacy | Yes | 12 | Yes |
| FG17P3P1F | Pharmacy | Yes | 6 | Yes |
| FG17P3P2F | Pharmacy | Yes | 4 | No |
| FG18M5P1F | Medicine | Yes | 8 | Yes |
| FG18M5P2F | Medicine | Yes | 5 | Yes |
| FG18M5P3F | Medicine | Yes | 8 | Yes |

*Longitudinal cases presented using pen portraits
